# Single-Nucleotide Polymorphisms Within Non-HLA Regions Are Associated With Engraftment Effectiveness for Patients With Unrelated Cord Blood Transplantation

**DOI:** 10.3389/fimmu.2022.888204

**Published:** 2022-06-13

**Authors:** Ding-Ping Chen, Tang-Her Jaing, Ai-Ling Hour, Wei-Tzu Lin, Fang-Ping Hsu

**Affiliations:** ^1^ Department of Laboratory Medicine, Linkou Chang Gung Memorial Hospital, Taoyuan, Taiwan; ^2^ Department of Medical Biotechnology and Laboratory Science, College of Medicine, Chang Gung University, Taoyuan, Taiwan; ^3^ Graduate Institute of Biomedical Sciences, College of Medicine, Chang Gung University, Taoyuan, Taiwan; ^4^ Department of Pediatrics, Division of Hematology/Oncology, Chang Gung Children’s Hospital, Chang Gung University, Taoyuan, Taiwan; ^5^ Department of Life Science, Fu Jen Catholic University, Taipei, Taiwan

**Keywords:** cord blood transplantation (CBT), single-nucleotide polymorphism (SNP), non-HLA genes, CTLA4, CD28, TNFSF4, PDCD1

## Abstract

Clinically, stem cells with matched human leukocyte antigens (HLAs) must be selected for allogeneic transplantation to avoid graft rejection. However, adverse reactions still occur after cord blood transplantation (CBT). It was inferred that the HLA system is not the only regulatory factor that may influence CBT outcomes. Therefore, we plan to investigate whether the single-nucleotide polymorphisms (SNPs) located in non-HLA genes are associated with the effectiveness of CBT. In this study, the samples of 65 donors from CBT cases were collected for testing. DNA sequencing was focused on the SNPs of non-HLA genes, cytotoxic T-lymphocyte-associated protein 4 (CTLA4), CD28, tumor necrosis factor ligand superfamily 4 (TNFSF4), and programmed cell death protein 1 (PDCD1), which were selected in regard to the literatures published in 2017 and 2018, which indicated that they were related to stem cell transplantation. Then, in combination with the detailed follow-up transplantation tracking database, these SNPs were analyzed with the risk of mortality, relapse, cytomegalovirus (CMV) infection, and graft-versus-host disease (GVHD). We found that there were 2 SNPs of CTLA4, 1 SNP of TNFSF4, and 2 SNPs of PDCD1 associated with the effectiveness of unrelated CBT. These statistically significant SNPs and haplotypes would be used in clinical to choose the best donor for the patient receiving CBT. Moreover, the polygenic risk scores (PRSs) with these SNPs could be used to predict the risk of CBT adverse reactions with an area under the receiver operating characteristic curve (AUC) of 0.7692. Furthermore, these SNPs were associated with several immune-related diseases or cancer susceptibility, which implied that SNPs play an important role in immune regulation.

## Introduction

Cord blood transplantation (CBT) is a type of hematopoietic stem cell transplantation (HSCT), which is an alternative treatment for blood disorders in children and adults (1–5). Two or less human leukocyte antigen (HLA) allele mismatches are acceptable in CBT (6, 7), which increases the opportunity of CBT in clinical situations. Unfortunately, even though HLA-matched cord blood stem cells are used, disease relapse and other post-transplant complications may still occur (8, 9). Based on the initial data analysis of CBT cases, we found that even though the HLA alleles were consistent between donor and recipient, the adverse events of post-CBT still existed. It was inferred that there were other important factors affecting the effectiveness of HSCT and CBT in addition to HLA genes. In the beginning, we focused on the genes that were surrounded by the classical HLA genes and followed the experimental methods shown by Petersdorf et al. (10). We observed that even if the HLA and HLA-related alleles between the donor and the recipient were consistent, several HSCT and CBT cases may still have poor outcomes (11). In recent years, the correlation between non-HLA genes and HSCT has been studied, including tumor necrosis factor ligand superfamily member 4 (TNFSF4; CD134) located on chromosome 1, and CD28, cytotoxic T-lymphocyte-associated protein 4 (CTLA4; CD152), and programmed cell death protein 1 (PDCD1; PD1) located on chromosome 2. TNFSF4 is a ligand of TNFRSF4 expressed on dendritic cells, B cells, and activated endothelial cells, which is involved in the immune response against infection. It is inferred that the incompatible locus of the TNFSF4 gene between donor and recipient may lead to transplant rejection through an immune mechanism (12). However, the correlation between the SNP of TNFSF4 and the outcomes of CBT still needs to be further explored at present. CD28, CTLA4, and PDCD1 involved in regulating T-cell activation play an important role in the immune response and as research hotspots (13–17). We speculated that the polymorphism of these genes would affect the prognosis of patients with CBT by interfering with immune regulation. In this study, we explored the association between the SNPs related to HSCT that were shown in published foreign literature and the engraftment effectiveness for patients with unrelated CBT in the Taiwan population. It was hoped to improve the effectiveness of post-CBT either by improving survival rate, reducing relapse rate, reducing the occurrence of graft-versus-host disease (GVHD), or reducing cytomegalovirus (CMV) infection.

## Methods and Materials

### Study Subjects

The research has been approved by the Institutional Review Board (IRB) of Chang Gung Memorial Hospital, with the ID 202101454B0. A total of 65 CBT cases signed the informed consent form. Additionally, the DNA sample of the donors corresponding to these 65 CBT cases was extracted for the follow-up DNA sequencing and SNP analysis. The clinical features and the diseases of patients are shown in **Table 1**.

**Table 1:** Characteristics of the patients recruited in this study.

	Number of patient (%) or median (range)		
Number of patients	65		
Median age in years at transplantation (range)	5 years old (27 days–15 years old)		
Male: Female	39 (60%): 26 (40%)		
Diagnosis		
Transfusion-dependent thalassemia	26	40.0%
Severe aplastic anemia	5	7.7%
Fanconi anemia	3	4.6%
ALL	7	10.8%
AML	3	4.6%
CML	1	1.5%
Inheritable disease	14	21.5%
(chronic primary granulomatous disease, X-linked severe combined immunodeficiency, Wiskott–Aldrich syndrome, malignant osteopetrosis)		
Tumor disease	6	9.2%
(neuroblastoma, retroperitoneal neuroblastoma, malignant tumor)		
HLA (HLA-A, -B, and -DRB1) compatibility		
Fully matched	15	23.1%
One mismatch	27	41.5%
Two mismatches	23	35.4%
ABO compatibility		
Full match	31	47.7%
Minor mismatch	18	27.7%
Major mismatch	16	24.6%
Survival	50	76.9%
GVHD		
Non-GVHD	11	16.9%
Grade 1–2	27	41.5%
Grade 3–4	14	21.5%
Chronic	13	20.0%
Cytomegalovirus (CMV) serology		
R-/D-	45	69%
R-/D+	20	31%
Conditioning regimen, *n* (%)			
Only chemotherapy (ACMI, CLIA, ECLIA)	30	46%	
Only radiotherapy (RIA)	1	2%	
Chemoradiotherapy	5	8%	
NA	29	45%	

In CMV, R, recipient; D, donor; +, seropositive; -, seronegative; NA, Not applicable.

### Definition of the Adverse Reaction of Post-CBT

Patients who are still alive at the end of the study are labeled as survivors. On the contrary, patients who died for whatever reason during the study are labeled as non-survivors. In the definition of CMV infection, the cytomegalovirus antigen or cytomegalovirus DNA detected in the patient’s body after transplantation is referred to as CMV infection. The definition of GVHD is based on the consensus of the International Blood and Marrow Transplant Research. Acute GVHD (aGVHD) that occurs within 100 days after transplantation is classified into 4 grades according to the clinical characteristics of the organ (18). Additionally, the grades of aGVHD are divided into 3 levels for analysis: grade I–II, grade III–IV, and absence of GVHD. Patients are said to have relapsed if they experienced one or more of the following situations: bone marrow morphology and minimal residual disease based on either flow cytometry, cytogenetics, imaging results, or short tandem repeat (STR) analysis. STR was analyzed by high-throughput amplicon sequencing (AmpFISTR Identifiler amplification kit, Thermo Fisher, Waltham, MA) according to the manufacturer’s instruction, which was used to evaluate the engraftments of posttranscription for identification of mixed chimerism (19). The definition of relapse was that the event was characterized by primary disease recurrence. The STR analyses supported the diagnosis of relapse as far as possible. The STR alleles in the chimeric test were used to monitor engraftment after transplantation. The presence of >5% recipient STR alleles in the chimeric test was defined as graft failure. Patients in continuous complete remission were censored at the last follow-up.

### Selection of SNPs

The 34 SNPs (CTLA4: rs11571315, rs733618, rs4553808, rs11571316, rs62182595, rs573554201, rs16840252, rs945677329, rs5742909, rs231775, rs56102377, rs56217811, rs55696217, rs231721, rs778932058, rs3087243, and rs11571319; TNFSF4: rs1234314, rs45454293, and rs181758110; CD28: rs1879877, rs3181096, rs3181097, rs3181098, rs28718975, rs28688913, rs28541784, rs20189072, and rs200353921; PDCD1: rs5839828, rs36084323, rs41386349, rs6705653, and rs2227982) were selected as sourced SNPs in this study because they had been reported to be associated with the risk of mortality, disease-free survival, transplant-related mortality, and relapse of acute or chronic GVHD in patients with HSCT.

### PCR and Sequencing

Three milliliters of the unlinked peripheral blood sample from the donors corresponding to the patients with CBT was collected, and the DNA was extracted by using the QIAamp DNA Blood Mini Kit (Qiagen, Valencia, California, USA). A total of 8 pairs of primers (**Table 2**) were used to amplify DNA fragments, covering 500 bp upstream and downstream of the sourced SNPs shown above. The PCR reaction volume was 25 μl, including 1 μl each of the forward and reverse primer, 8 μl of Hot Start Taq DNA polymerase (Agilent, Santa Clara, California, USA), 1 μg of sample DNA, and 14 μl of double-distilled water (ddH_2_O). The PCR program began at 94°CC for 4 min, 30 cycles of 94°CC for 30 s, 58°CC for 30 s, and 72°CC for 45 s, and then 72°CC for 10 min. Subsequently, 1.5%–2% agarose gel was used to analyze the PCR products through gel electrophoresis. According to the manufacturer’s instructions, the Big Dye Terminator Cycle Sequencing kit (Thermo Fisher, Waltham, Massachusetts, USA) and the ABI PRISM genetic analyzer (Thermo Fisher, Waltham, Massachusetts, USA) were used for direct sequencing. Because of the deficiency of genomic DNA and failure of PCR reaction, the SNP data could not be completed.

**Table 2:** Primer sequences used for PCR and DNA sequencing.

Gene name	Genomic region	Primer sequence	PCR product (bp)
CTLA4	Promoter	F: 5’-GGCAACAGAGACCCCACCGTT-3’ R: 5’-GAGGACCTTCCTTAAATCTGGAGAG-3’	1,233
	Promoter and exon 1	F: 5’-CTCTCCAGATTTAAGGAAGGTCCTC-3’ R: 5’-GGAATACAGAGCCAGCCAAGCC-3’	1,169
	Exon 4	F: 5’-CTAGGGACCCAATATGTGTTG-3’ R: 5’-AGAAACATCCCAGCTCTGTC-3’	1,039
	Exon 4 and 3’-UTR	F: 5’-GCTTGGAAACTGGATGAGGTCATAGC-3’ R: 5’-AGAGGAAGAGACACAGACAGAGTTGC-3’	1,204
TNFSF4	Promoter and exon 1	F: 5’-GGCTTGGAGTCTATGATATTGTGCC-3’ R: 5’-GAAGGGCGTTTAACCACACTTTACG-3’	1,725
CD28	Promoter and exon 1	F: 5’-GGGTGGTAAGAATGTGGATGAATC-3’ R: 5’-CAAGGCATCCTGACTGCAGCA-3’	1,961
PDCD1	Promoter and exon 1	F: 5’-ACCCACACAGCCTCACATCTCT-3’ R: 5’-AAACTGAGGGTGGAAGGTCCCT-3’	1,778
	Exon 4, intron 4, and exon 5	F: 5’-TGGTGACCCCAAGTGTGTTTCTC-3’ R: 5’-GAGGAATTTTTCACCGGAGGGC-3’	2,234

F, forward primer; R, reverse primer.

### Statistical Analysis

Chi-square test and Fisher’s exact test were used to analyze the difference of donor genotype frequency in each outcome of the recipient receiving CBT (mortality, relapse, CMV infection, and GVHD) through dominant (AA vs. Aa +aa), recessive (AA +Aa vs. aa), and additive (AA vs. Aa vs. aa) models. In the analysis model, “A” refers to the allele with a higher frequency, and “a” refers to the allele with a lower frequency in the subjects. The Kaplan–Meier method and log rank test were used for survival analysis. The overall survival (OS) analysis was based on the mortality-related SNPs. Event-free survival (EFS) was based on the CMV-related, GVHD-related, and relapse-related SNPs, where the censoring point was the time point of patient death or the specific event occurrence. Moreover, we further analyzed the impact of these events on survival time. After that, the Kolmogorov–Smirnov test was used for testing normality of OS and EFS distribution. Multivariate analysis with post-hoc test and Tukey test were used to verify the impact of SNP on the prognosis. These analyses were analyzed by SPSS 17.0. *D*’ and the *p*-value of linkage disequilibrium (LD) were analyzed by genetics package with R, and the figures of LD were generated by using HaploView 4.2 (https://www.broadinstitute.org/haploview/haploview). Moreover, we made correction for multiple comparisons by using Bonferroni correction. Thus, *p* = 0.0012 (0.05/42SNPs) was referred to as the significance level in this study. To create a model to predict the failure of CBT, we firstly calculated the polygenic risk scores (PRSs) by referring to Mavaddat et al. (20) and made a receiver operating characteristic (ROC) curve to evaluate the predictive ability of PRSs for predicting risk of CBT failure by referring to Wang et al. (21). The area under the ROC curve (AUC) was calculated by the pROC package (https://web.expasy.org/pROC/).

## Results

The tracking data of the patients who received CBT and the SNP data of the donor corresponding to the patient were combined to find out the association between SNP and engraftment effectiveness. In the SNP analysis, the significance standard was set as *p* = 0.0012. In CTLA4 gene analysis, we found that one SNP located in the promoter region and 1 SNP located in the 3’UTR had statistical significance. In CD28 gene analysis, no significant SNP was found. In TNFSF4 gene analysis, there was 1 statistically significant SNP located in the promoter region. In PDCD1 gene analysis, 2 SNPs were statistically significant. The significance standard was set as *p* = 0.05 in Kaplan–Meier analysis, multivariate analysis, and haplotype analysis.

### Association Between the Outcomes of Post-CBT and the SNPs

In SNP analysis (**Tables 3**–**6**), only one SNP located in the TNFSF4 gene was associated with the outcomes of post-CBT. The genotype of rs1234314 was associated with the rate of death (*p* < 0.001). Additionally, when the donor had a CC genotype, the probability of death for the recipient was increased 15.4 times (*p* < 0.001, 95% CI = 3.309–71.675) (**Table 5**).

**Table 3:** The SNPs in the CTLA4 gene associated with the outcomes of post-CBT.

SNP	Gene position	No. of patients (%)			Model	Logistic regression *p*	OR
GVHD I–II							
rs733618	CTLA4 Promoter	CC	CT	TT	Additive	0.002*	N/A
Case		13 (86.7%)	1 (16.7%)	11 (84.6%)	Dominant	0.240	0.264 (0.046–1.528)
Control		2 (13.3%)	5 (83.3%)	2 (15.4%)	Recessive	0.427	2.750 (0.474–15.964)
GVHD							
rs231775	CTLA4 Exon 1	GG	AG	AA	Additive	0.054	N/A
Presence		14 (66.7%)	26 (92.9%)	7 (87.5%)	Dominant	0.028	5.500 (1.240–24.404)
Absence		7 (33.3%)	2 (7.1%)	1 (12.5%)	Recessive	1.000	1.575 (0.172–14.452)
GVHD I–II							
rs3087243	CTLA4 3’-UTR	GG	AG	AA	Additive	0.015	N/A
Case		9 (28.1%)	15 (60%)	1 (100%)	Dominant	0.007*	16.000 (1.735–147.5)
Control		9 (50%)	1 (6.3%)	0 (0%)	Recessive	1.000	N/A
GVHD							
rs3087243	CTLA4 3’-UTR	GG	AG	AA	Additive	0.051	N/A
Absence		23 (71.9%)	24 (96%)	1 (100%)	Dominant	0.017	9.783 (1.148–83.329)
Presence		9 (28.1%)	1 (4%)	0 (0%)	Recessive	1.000	N/A

N/A, Not applicable. Additive model: AA vs. Aa vs. aa; Dominant model: AA vs. Aa +aa; Recessive model: AA +Aa vs. aa, where “A” refers to a major allele, and “a” is a minor allele. “*” represents statistical significance (p < 0.0125). Case: the patients with the status. Control: the patients without the status.

**Table 4:** The SNPs in the CD28 gene associated with the outcomes of post-CBT.

SNP	Gene position	No. of patients (%)			Model	Logistic regression *p*	OR
**Mortality**							
rs3181097	CD28 Promoter	GG	AG	AA	Additive	0.061	N/A
Case		3 (17.6%)	4 (12.9%)	4 (50%)	Dominant	1.000	0.830 (0.191–3.609)
Control		14 (82.4%)	27 (87.1%)	4 (50%)	Recessive	0.040*	0.171 (0.034–0.846)
**Relapse**							
rs28718975	CD28 Promoter	TT	CT	CC	Additive	0.018*	N/A
Case		29 (67.4%)	8 (88.9%)	0 (0%)	Dominant	1.000	0.966 (0.248–3.759)
Control		14 (32.6%)	1 (11.1%)	3 (100%)	Recessive	0.031*	N/A
**Relapse**							
rs28688913	CD28 Promoter	CC	CT	TT	Additive	0.018*	N/A
Case		29 (67.4%)	8 (88.9%)	0 (0%)	Dominant	1.000	0.966 (0.248–3.759)
Control		14 (32.6%)	1 (11.1%)	3 (100%)	Recessive	0.031*	N/A
**Relapse**							
rs28541784	CD28 Promoter	CC	CT	TT	Additive	0.018*	N/A
Case		29 (67.4%)	8 (88.9%)	0 (0%)	Dominant	1.000	0.966 (0.248–3.759)
Control		14 (32.6%)	1 (11.1%)	3 (100%)	Recessive	0.031*	N/A
**GVHD I–II**							
rs3181096	CD28 Promoter	CC	CT	TT	Additive	0.042*	N/A
Case		6 (46.2%)	12 (80%)	5 (100%)	Dominant	0.026*	6.611 (1.280–34.14)
Control		7 (53.8%)	3 (20%)	0 (0%)	Recessive	0.291	N/A

N/A, Not applicable. Additive model: AA vs. Aa vs. aa; Dominant model: AA vs. Aa +aa; Recessive model: AA +Aa vs. aa, where “A” refers to a major allele, and “a” is a minor allele. “*” is represented to the statistical significance (p<0.0125) Case: the patients with the status. Control: the patients without the status.

**Table 5:** The SNPs in the TNFSF4 gene associated with the outcomes of post-CBT.

SNP	Gene position	No. of patients (%)			Model	Logistic regression *p*	OR
Mortality							
rs1234314	TNFSF4 promoter	GG	CG	CC	Additive	<0.001*	N/A
Case		3 (17.6%)	2 (6.3%)	7 (63.6%)	Dominant	<0.001*	15.4 (3.309–71.675)
Control		14 (82.4%)	30 (93.8%)	4 (36.4%)	Recessive	1	1.235 (0.291–5.252)

N/A, Not applicable. Additive model: AA vs. Aa vs. aa; Dominant model: AA vs. Aa +aa; Recessive model: AA +Aa vs. aa, where “A” refers to a major allele, and “a” is a minor allele. “*”represents statistical significance (p < 0.0125). Case: the patients with the status. Control: the patients without the status.

**Table 6:** The SNPs in the PDCD1 gene associated with the outcomes of post-CBT.

SNP	Gene position	No. of patients (%)			Model	Logistic regression *p*	OR
Mortality							
rs10204525	PDCD1 Promoter	TT	CT	CC	Additive	0.052	N/A
Case		10 (33.3%)	1 (4.8%)	2 (25%)	Dominant	1.000	1.212 (0.214–6.864)
Control		20 (66.7%)	20 (95.2%)	6 (75%)	Recessive	0.033	0.231 (0.056–0.951)
Relapse							
rs36084323	PDCD1 Promoter	CC	CT	TT	Additive	0.031	N/A
Case		9 (45%)	22 (81.5%)	9 (69.2%)	Dominant	0.012*	4.210 (1.331–13.320)
Control		11 (55%)	5 (18.5%)	4 (30.8%)	Recessive	1.000	1.161 (0.309–4.362)
Relapse							
rs2227982	PDCD1 Exon 5	GG	AG	AA	Additive	0.016	N/A
Case		8 (42.1%)	24 (80%)	10 (76.9%)	Dominant	0.004*	5.194 (1.612–16.739)
Control		11 (57.9%)	6 (20%)	3 (23.1%)	Recessive	0.520	1.771 (0.429–7.311)

N/A, Not applicable. Additive model: AA vs. Aa vs. aa; Dominant model: AA vs. Aa +aa; Recessive model: AA +Aa vs. aa, where “A” refers to a major allele, and “a” is a minor allele. “*” represents statistical significance (p < 0.0125). Case: the patients with the status. Control: the patients without the status.

### Kaplan–Meier Analysis of Overall Survival/Event-Free Survival Based on the Outcome-Related SNPs

**Figure 1:**
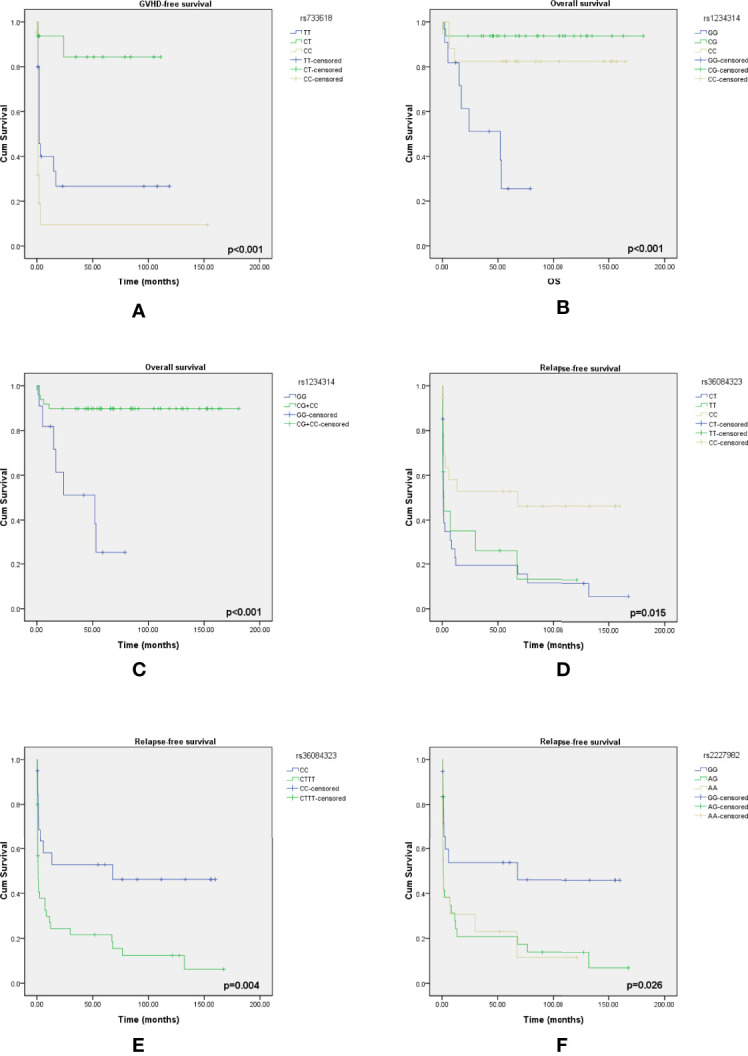
Kaplan–Meier curves of overall survival with statistical significance based on the mortality-related SNPs and event-free survival based on the GVHD-related SNPs and relapse-related SNPs. **(A)** The survival time had a significant difference between the 3 genotypes of rs733618. **(B)** The survival time had a significant difference between the 3 genotypes of rs1234314. **(C)** The survival time had a significant difference between the 2 genotype groups of rs1234314. **(D)** The survival time had a significant difference between the 3 genotypes of rs36084323. **(E)** The survival time had a significant difference between the 2 genotype groups of rs36084323. **(F)** The survival time had a significant difference between the 3 genotypes of rs2227982.

According to the results of the log-rank test, we found that the rs733618 of CTLA4, the rs1234314 of TNFSF4, and the rs36084323 and rs2227982 of PDCD1 were associated with OS and EFS. In **Figure 1A**, the GVHD-free survival time was significantly different between 3 genotypes (CC, CT, and TT) of rs733618 (*p* < 0.001). The estimated mean survival time of the patient was 34.92 months ( ± 12.46 SD) when the donor had a TT genotype, the estimated mean survival time was 95.97 months ( ± 9.88 SD) when the donor had a CT genotype, and the estimated mean survival time was 15.75 months ( ± 12.45 SD) when the donor had a CC genotype. The average survival time was 58.68 months. The OS time was significantly different between the 3 genotypes (GG, CG, and CC) of rs1234314 (*p* < 0.001). The estimated mean survival time of the patient was 39.99 months ( ± 9.08 SD) when the donor had a GG genotype, the estimated mean survival time was 169.81 months ( ± 7.66 SD) when the donor had a CG genotype, and the estimated mean survival time was 136.88 months ( ± 14.74 SD) when the donor had a CC genotype. The average survival time was 146.40 months (**Figure 1B**). Moreover, the OS time of rs1234314 also had significance based on the dominant model (GG vs. CG +CC, *p* < 0.001). The estimated mean survival time was 39.99 months ( ± 9.08 SD) when the donor had a GG genotype, and the estimated mean survival time was 162.96 months ( ± 7.65 SD) when the donor had a CG +CC genotype. The average survival time was 146.40 months (**Figure 1C**). The relapse-free survival time was significantly different between the 3 genotypes (CC, CT, and TT) of rs36084323 (*p* = 0.015). The estimated mean survival time of the patient was 79.83 months ( ± 17.59 SD) when the donor had a CC genotype, the estimated mean survival time was 28.51 months ( ± 12.73 SD) when the donor had a TT genotype, and the estimated mean survival time was 25.05 months ( ± 9.74 SD) when the donor had a CT genotype. The average survival time was 46.00 months (**Figure 1D**). Moreover, the relapse-free survival time of rs36084323 also had significance based on the dominant model (CC vs. CT +TT, *p* = 0.004). The estimated mean survival time was 79.83 months ( ± 17.59 SD) when the donor had a CC genotype, and the estimated mean survival time was 27.61 months ( ± 8.41 SD) when the donor had a CT +TT genotype (**Figure 1E**). The relapse-free survival time was significantly different between the 3 genotypes (GG, AG, and AA) of rs2227982 (*p* = 0.026). The estimated mean survival time of the patient was 79.75 months ( ± 18.68 SD) when the donor had a GG genotype, the estimated mean survival time was 28.17 months ( ± 9.84 SD) when the donor had a AG genotype, and the estimated mean survival time was 25.03 months ( ± 11.61 SD) when the donor had a AA genotype. The average survival time was 44.46 months (**Figure 1F**). These significant SNPs were also associated with the outcomes of post-bone marrow transplantation (BMT) in our previous study (22), which was summarized in **Table 7**.

**Table 7:** Summarized the SNPs that were associated with adverse outcomes of post-BMT and post-CBT.

Gene	SNP	Transplantation method	Outcome
TNFSF4 (−738) promoter	rs1234314	BMT (AML) CBT	GVHD III–IV mortality
CD28 (−891) promoter	rs28541784	BMT (ALL) CBT	Chronic GVHD relapse
CTLA4 (+49) exon-1	rs231775	BMT (ALL) BMT (ALL) CBT	GVHD I–II Chronic GVHD GVHD
CTLA4 (ct60) 3’UTR	rs3087243	BMT (AML) CBT CBT	mortality GVHD I–II GVHD
PDCD1 (−606) promoter	rs36084323	BMT (ALL) BMT (ALL) CBT	CMV infection relapse relapse
PDCD1 (+699) exon-5	rs2227982	BMT (AML) BMT (ALL) BMT (ALL) BMT (ALL) CBT	CMV infection relapse GVHD I–II Chronic GVHD relapse

### Association Between the Outcomes of Post-CBT and Overall Survival and Event-Free Survival

The *p*-value of OS from the Kolmogorov–Smirnov test was 0.338, which meant that the OS had a normal distribution. However, the *p*-value of CMV-free survival, GVHD-free survival, and relapse-free survival had a non-normal distribution (*p* = 0.040, <0.001, and <0.001, respectively). Then, the independent *t*-test and Kruskal–Wallis test were used to analyze the difference of survival time between presence and absence of events. The data are summarized in **Table 8**. There were 15 patents (23%) who died, 20 patients (31%) with CMV infection, 54 patients (83%) with GVHD, and 44 patients (68%) with relapse in this study. Moreover, the patients with GVHD 3–4 would affect the CMV-free survival (*p* = 0.028) and OS (*p* = 0.038). Additionally, the patients with mortality and with or without relapse were associated with CMV-free survival (*p* < 0.001 and *p* = 0.005, respectively), and the patients with or without relapse were also associated with OS (*p* = 0.043).

**Table 8:** Analysis of the association between events and overall survival and event-free survival.

	Overall survival		Non-relapse survival	CMV-free survival	GVHD-free survival
	*n*	*p*	*p*	*p*	*p*
Survival	50				
mortality	15	NA	0.709	<0.001*	0.076
CMV-infection	20				
Non CMV-infection	45	0.185	0.546	NA	0.239
GVHD	54				
Non GVHD	11	0.073	0.248	0.083	NA
GVHD1-2	27				
Non GVHD	11	0.236	0.326	0.227	NA
GVHD3-4	14				
Non GVHD	11	0.038*	0.324	0.028*	NA
Chronic GVHD	13				
Non GVHD	11	0.091	0.310	0.212	0.173
Relapse	44				
Non relapse	21	0.043*	NA	0.005*	0.073

“*” represents statistical significance with p-value <0.05. NA, Not Applicable.

### Multivariate Analysis for Verifying the Impact of SNPs on the Prognosis of CBT

Because there were many factors that would affect the prognosis of CBT, including disease type and HLA compatibility, we took HLA compatibility and disease type as covariates in a multivariate analysis to exclude the interference of these two factors. All the *p*-values of Bartlett’s test were less than 0.05. According to the data (**Table 9**), we found that the rs733618 (*p* = 0.006), rs1234314 (*p* = 0.001), rs36084323(*p* = 0.050), and rs2227982 (*p* = 0.013) had significance in Wilks’ Lambda. After the post-hoc test, we found that the survival time of GVHD-EFS was significantly different between the homozygote genotype group and the heterozygote genotype group of rs733618 (CC vs. CT, *p* < 0.001; TT vs. CT, *p* = 0.006). The survival time of OS was significantly different between the GG genotype and other genotypes of rs1234314 (GG vs. CG, *p* = 0.011; GG vs. CC, *p* = 0.014). The survival time of CMV-EFS was significantly different between the GG genotype and the CC genotype of rs1234314 (*p* = 0.013). Additionally, the survival time of OS and CMV-EFS had significance in rs1234314 based on the dominant model (*p* = 0.002 and 0.015, respectively). The survival time of relapse-EFS was significantly different between the CC genotype and CT genotype of rs36084323 (*p* = 0.025). Moreover, it was significant in relapse-EFS based on the dominant model (CC vs. CT +TT), *p* = 0.023. In another relapse-related SNP of PDCD1, rs2227982, the survival time of OS was significantly different between the GG genotype and the AG genotype (*p* = 0.025) and AG +AA (*p* = 0.025).

**Table 9:** Summarized the significant results of log rank test, Wilks’ Lambda, and *post-hoc* test.

SNP	Status	Model	Log rank *p*	*F*	Wilks’ Lambda *p*-value	EFS	Genotype	Tukey HSD *p*-value
CTLA4								
rs733618	GVHD1-2	Additive	<0.001*	4.072	0.006*	GVHD-EFS	TT vs. CT	0.006*
							CC vs. CT	<0.001*
CD28								
rs3181097	Mortality	Recessive	0.027*	0.092	2.266			
TNFSF4								
rs1234314	Mortality	Additive	<0.001*	4.416	0.001*	OS	GG vs. CG	0.011*
							GG vs. CC	0.014*
						CMV-EFS	GG vs. CC	0.013*
		Dominant	<0.001*	1.251	0.301	OS	GG vs. CG+CC	0.002*
PDCD1						CMV-EFS	GG vs. CG+CC	0.015*
rs10204525	Mortality	Recessive	0.028*	2.131	0.107			
rs36084323	Relapse	Additive	0.015*	1.634	0.124			
		Dominant	0.004*	2.548	0.050*	Relapse-EFS	CC vs. CT+TT	0.023*
rs2227982	Relapse	Additive	0.026*	1.814	0.082			
		Dominant	0.007*	3.468	0.013*	OS	GG vs. AG+AA	0.025*

Tukey HSD, Tukey honestly significant difference. “*” represents statistical significance with p-value <0.05.

### Haplotype Analysis in Each Event

The SNPs with *p* < 0.05 from each event analysis (**Tables 3**–**6**) were collected into a haplotype for analysis. We found that the A_rs231775_A_rs3087243_ haplotype was associated with GVHD (*p* = 0.034). When the donor had an A-allele in both rs231775 and rs3087243, the CBT cases would have a higher risk to get GVHD (OR = 8.625, 95% CI = 1.011–73.578). Among them, these two SNPs were all located in the CTLA4 gene. Moreover, the G_rs3181097_C_rs10204525_ haplotype was associated with mortality (*p* = 0.019). When the donor had a G-allele in rs3181097 and a C-allele in rs10204525, the CBT cases would have a lower mortality rate (OR = 0.104, 95% CI = 0.012–0.874) (**Table 10**). In relapse analysis, there was no sample with T_rs28718975_C_rs28688913_C_rs28541784_A_rs2227982_T_rs36084323_, and there was no significant haplotype in GVHD1-2 analysis.

**Table 10:** The significant haplotypes associated with events.

Events	Haplotype	OR	95% CI	*p*-value
GVHD	Ars231775Ars3087243	8.625	1.011–73.578	0.034
Mortality	Grs3181097Crs10204525	0.104	0.012–0.874	0.019

### The Model to Predict CBT Failure

**Figure 2:**
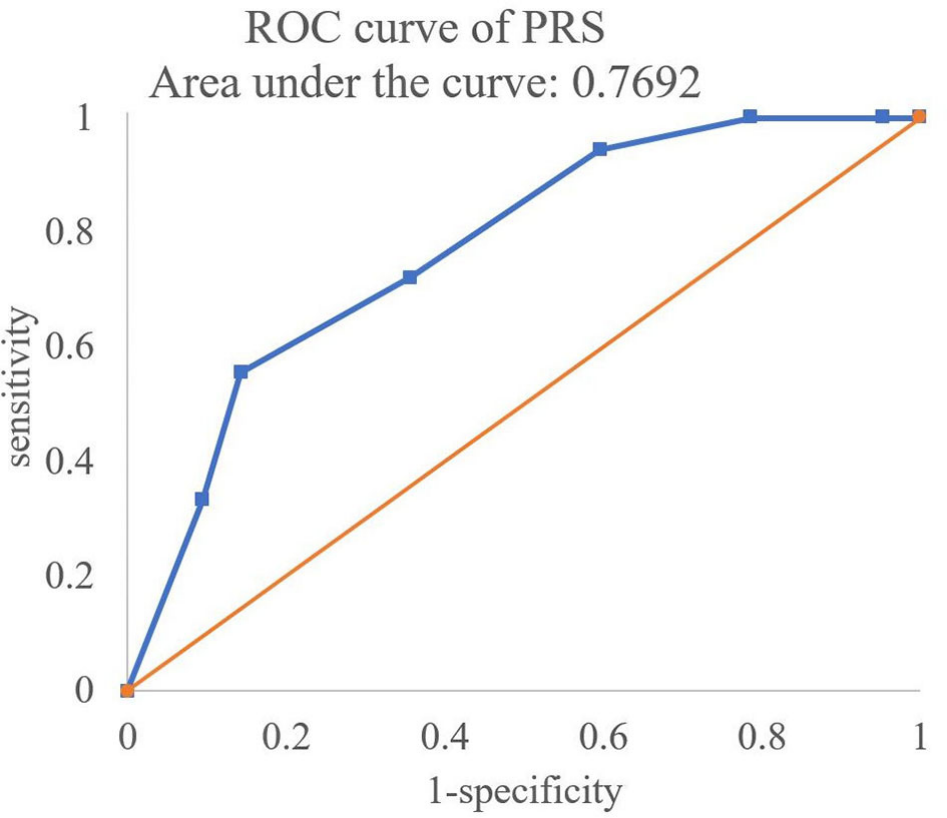
The ROC curve of the PRS for predicting the risk of CBT adverse reactions.

First, the PRSs with the OR of significant SNPs (*p* < 0.05) in **Tables 3**–**6** were calculated. The formula of PRS is shown in ref. (20). The logarithm of OR was regarded as the coefficient of PRS. If the OR was NA, the coefficient was 0. Multiply this coefficient by the number of risk alleles (0,1,2) in each SNP and then add these data and divide the sum by the number of SNPs to calculate the PRSs, in which the number of SNPs less than 10 were excluded. Then, the ROC curve of the PRSs was created to predict the failure risk of CBT with an AUC of 0.7692 (**Figure 2**).

## Discussion

In this study, we explored the association between the SNPs (TNFSF4, CTLA4, CD28, and PDCD1) from the corresponding donor and the outcomes (mortality, relapse, CMV infection, and GVHD) of post-CBT from the participant. The data showed that there were 5 SNPs in these four genes associated with the outcomes of post-CBT. Integrating the research results of the literature published in the *Frontiers in Immunology* journal in 2021 by our team that explored the association between immune regulatory gene polymorphism and the outcomes of post-HSCT (22), it was found that the rs1234314 of TNFSF4, the rs36084323 and rs2227982 of PDCD1, and the rs733618 and rs3087243 of CTLA4 were also associated with the outcomes of post-bone marrow transplantation (BMT) in adults in addition to the CBT case in children. These SNPs affected mortality, GVHD, and relapse in both BMT cases and CBT cases. The patients receiving BMT were mostly adults and with acute leukemia, and the patients receiving CBT were children and the half were non-malignant. These SNPs affect GVHD, relapse, and mortality in the different settings of graft source and disease type. This indicated that these SNPs play an important role in the immunity of transplantation. However, correlation does not imply causation. Thus, in the future, the biological function of these SNPs should be further discussed to clarify the mechanism. Then, in the survival analysis, it was found that the rs733618 of the CTLA4 gene, the rs1234314 of the TNFSF4 gene, and the rs36084323 and rs2227982 of the PDCD1 gene had a significant impact on the OS or EFS of CBT patients. In addition, after excluding the influence of HLA compatibility and disease type on prognosis, it was found that the specific genotype of rs733618, rs1234314, rs36084323, and rs2227982 would affect the survival time of patients.

CTLA4 is known as an immune checkpoint, which plays a vital role in the process of immune regulation. In the process of T-cell activation, CD28 interacts with CD80/CD86 expressed on the antigen-presenting cells and provides the stimulatory signal to promote T-cell activation after the T-cell receptor recognizes the antigen. Then, CTLA4 will be expressed on the activated T cells and compete with CD28 for CD80/CD86 to avoid T-cell over-activation through inhibiting cell proliferation and degradation to block the downstream pathways of T-cell activation (23, 24). rs733618 is located in the promoter region of CTLA4. Multiple myeloma patients receiving bortezomib-based regimens with the rs733618 CC genotype had significantly lower disease-free survival and OS than those with CT +TT (25). Our data showed that the CC genotype of rs733618 from a donor had the worst GVHD-free survival for the patient with CBT. This indicated that the CC genotype of rs733618 had an adverse effect on the outcome of transplantation in patients or donors. Wang et al. indicated that the variant in rs733618 would affect the transcription activity of Nuclear Factor I and c/EBPβ, leading to the decreased expression level of CTLA4. Moreover, the T-to-C mutation in this position may interfere with alternative splicing and thus decreased the function of CTLA4 (26). It was suggested that rs733618 would affect the poor outcomes of transplantation by decreasing the expression level and function of CTLA4.

The TNFSF4 gene is a member of the tumor necrosis factor superfamily protein, which is the key to the coordination of innate or adaptive immune cells. The interaction between TNFSF4 and its ligand, OX40, plays an important role in the pathogenesis of autoimmune diseases and cancers (27). A literature indicated that the CC genotype of rs1234314 provided protective effects against allergic rhinitis in the Chinese Han population (28). However, in our research results, the probability of death for the recipient receiving CBT and the probability of severe GVHD for the recipient receiving BMT would increase when the donor had a CC genotype in rs1234314. This meant that this SNP may play a different role in autoimmune disease and the prognosis of CBT and BMT. However, the biological function of rs1234314 is not clear and, thus, needs to be verified in the future.

The role of PD1 is similar to CTLA4. They are involved in the regulation of T-cell activation through inhibiting the inflammatory activity of T cells to produce immune tolerance. This regulatory mechanism can prevent autoimmune diseases, but it may also prevent the immune system from killing cancer cells, leading to recurrence (29, 30). The rs36084323 located in the promoter region and rs2227982 located in the exon 5 region of PDCD1 were associated with the outcomes of childhood CBT and adult BMT. rs36084323 had been shown to be associated with several cancers, such as breast cancer, epithelial ovarian cancer, and esophageal cancer, and it was also associated with several immune-related diseases, such as rheumatoid arthritis and pregnancy losses (31). da Silva indicated that rs36084323 with an A-allele would increase the expression level of PDCD1 (32). Therefore, rs36084323 related to poor outcomes of post-CBT may have affected the mRNA expression of PDCD1. In addition, the polymorphism of rs2227982 was associated with breast cancer, ankylosing spondylitis, multiple myeloma, and chronic HBV infection, among others (31). The rs2227982 G-to-A mutation will lead to the substitution of alanine for valine, which may lead to a change in the structure and function of PD1 and further influence the poor prognosis of transplantation and progression of diseases (33). Therefore, whether the amino acid change would affect the PD1 protein structure or its function needs to be further explored.

Moreover, a recent literature showed that the clinical outcomes after kidney transplantation were highly variable between individuals, which was related to the gene polymorphism in the B-cell activating factor system. The authors indicated that the specific SNP was important to the prognosis of kidney transplantation. It could inform future personalized/precision medicine efforts and functional genomic studies in patients with kidney transplantation (34). Similarly, the findings in this study will provide a foundation of personalized medicine for CBT patients. In addition, we also made a model for predicting the risk of CBT adverse reaction by PRSs. When the AUC is higher than 0.5, it means that the prediction model is better than random guessing and has a certain predictive ability. The AUC in the present study was 0.7692, which indicated that the predictive abilities of the PRSs with the significant SNPs (*p* < 0.05) were available.

In summary, the non-HLA gene polymorphism of donors could be used as an indicator for predicting the prognosis of CBT. Therefore, when selecting the best graft for CBT, the SNPs of non-HLA genes must be considered in addition to the consistency of HLA alleles. The haplotypes A_rs231775_A_rs3087243_ and G_rs3181097_C_rs10204525_ could be used as the basis for selecting the most suitable donor. Moreover, the PRSs with the significant SNPs in this study could be used to predict the risk of CBT adverse reaction. Furthermore, these SNPs that intersect in CBT and BMT studies may be the hub of immune mechanisms. By affecting the immune response from the origin, they could cause a variety of immune-related disorders or adverse outcomes after transplantation.

## Data Availability Statement

The datasets presented in this study can be found in online repositories. The names of the repository/repositories and accession number(s) can be found in the article/**Supplementary Material**.

## Ethics Statement

The research has been approved by the Institutional Review Board (IRB) of Chang Gung Memorial Hospital, with the ID 201901759B0, 202101454B0. The patients/participants provided their written informed consent to participate in this study.

## Author Contributions

D-PC conceived and designed the experiments, wrote and reviewed the final draft. T-HJ contributed the materials. F-PH and A-LH performed the experiments and analyzed and interpreted data. W-TL wrote the draft of the manuscript. All authors contributed to the article and approved the submitted version.

## Funding

This study was supported by grants to D-PC from the Ministry of Science and Technology (109-2320-B-182A-012). The funders had no role in study design, data collection and analysis, decision to publish, or preparation of the manuscript.

## Conflict of Interest

The authors declare that the research was conducted in the absence of any commercial or financial relationships that could be construed as a potential conflict of interest.

## Publisher’s Note

All claims expressed in this article are solely those of the authors and do not necessarily represent those of their affiliated organizations, or those of the publisher, the editors and the reviewers. Any product that may be evaluated in this article, or claim that may be made by its manufacturer, is not guaranteed or endorsed by the publisher.
