# Supplementary material for: Single-Nucleotide Polymorphisms Within Non-HLA Regions Are Associated With Engraftment Effectiveness for Patients With Unrelated Cord Blood Transplantation
Source: Front Immunol. 2022 Jun 13;13:888204. doi: 10.3389/fimmu.2022.888204 (PMC9234117; doi:10.3389/fimmu.2022.888204)
Supplement: Supplementary file 1 [file DataSheet_1.docx]

Single nucleotide polymorphisms within non-HLA regions are associated with engraftment effectiveness for patients with unrelated cord blood transplantation

**local_identifier NCBI_subsnp#**

rs181758110_2 2137544327

rs45454293 5799401603

rs1234314 5799401604

rs147669352 5799401605

rs1879877 5799401606

rs3181096 5799401607

rs3181097 5799401608

rs3181098 5799401609

rs28718975 5799401610

rs28688913 5799401611

rs28541784 5799401612

rs201801072 5799401613

rs200353921 5799401614

rs11571315 5799401615

rs733618 5799401616

rs4553808 5799401617

rs11571316 5799401618

rs62182595 5799401619

rs573554201 5799401620

rs16840252 5799401621

rs945677329 5799401622

rs5742909 5799401623

rs231775 5799401624

rs56102377 5799401625

rs56217811 5799401626

rs1581575882 5799401627

rs55696217 5799401628

rs231721 5799401629

rs778932058 5799401630

rs3087243 5799401631

rs11571319 5799401632

rs10204525 5799401633

rs1331108508 5799401634

rs56029561 5799401635

rs2227981 5799401636

rs2227982 5799401637

rs6705653 5799401638

rs41386349 5799401639

rs11568821 5799401640

rs36084323 5799401641

rs5839828 5799401642

rs944761632 5799401643
