# Supplementary material for: Single-Nucleotide Polymorphisms Within Non-HLA Regions Are Associated With Engraftment Effectiveness for Patients With Unrelated Cord Blood Transplantation
Source: Front Immunol. 2022 Jun 13;13:888204. doi: 10.3389/fimmu.2022.888204 (PMC9234117; doi:10.3389/fimmu.2022.888204)
Supplement: Supplementary file 2 [file DataSheet_2.docx]

**Supplementary Figures**


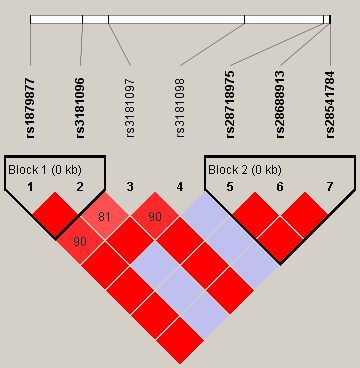


Supplementary Figure 1. The linkage disequilibrium (LD) analysis of the SNPs in CD28 gene. The number in the boxes was D’ value, which was measured between the pair of these 7 SNPs. The red color in the boxes means the two SNPs have high linkage; the closer to white color means that the linkage decreases gradually; the light purple color means they absolutely have no linkage. There were two blocks shown in CD28 gene, and the block was defined as it scarcely had evidence for historical recombination in this region (Gabriel SB, Science, 2002).


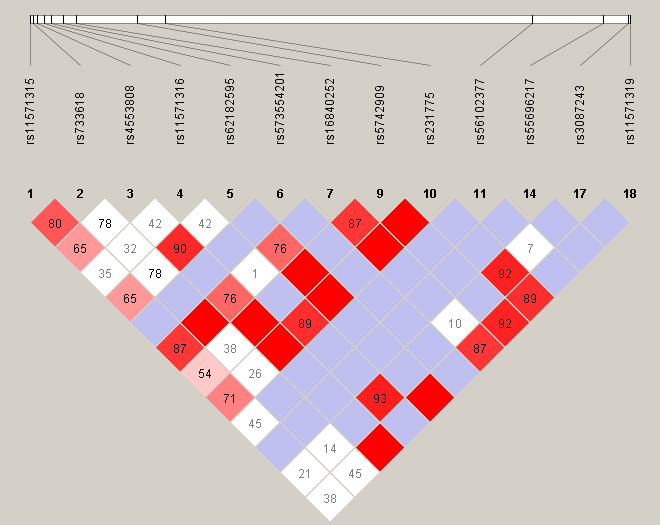


Supplementary Figure 2. The linkage disequilibrium (LD) analysis of the SNPs in CTLA4 gene. The number in the boxes was D’ value, which was measured between the pair of these 13 SNPs. The red color in the boxes means the two SNPs have high linkage; the closer to white color means that the linkage decreases gradually; the light purple color means they absolutely have no linkage. There were no block shown in CTLA4 gene.


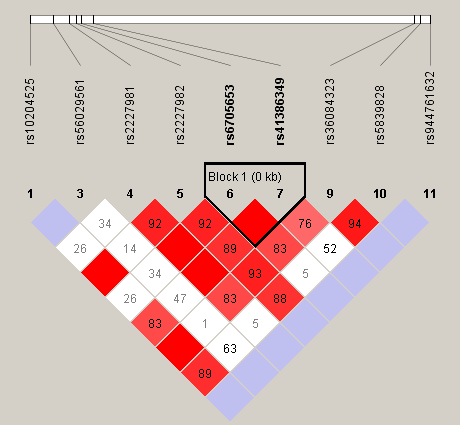


Supplementary Figure 3. The linkage disequilibrium (LD) analysis of the SNPs in PDCD1 gene. The number in the boxes was D’ value, which was measured between the pair of 9 SNPs. The red color in the boxes means the two SNPs have high linkage; the closer to white color means that the linkage decreases gradually; the light purple color means they absolutely have no linkage. There was one block shown in PDCD1 gene, and the block was defined as it scarcely had evidence for historical recombination in this region (Gabriel SB, Science, 2002).


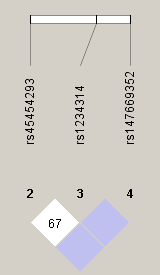


Supplementary Figure 4. The linkage disequilibrium (LD) analysis of the SNPs in TNFSF4 gene. The number in the boxes was D’ value, which was measured between the pair of 3 SNPs. The red color in the boxes means the two SNPs have high linkage; the closer to white color means that the linkage decreases gradually; the light purple color means they absolutely have no linkage. There was no block shown in TNFSF4 gene.
